# The use of confocal Raman microscopy and microfluidic channels to monitor the location and mobility of β-carotene incorporated in droplet-stabilized oil-in-water emulsions

**DOI:** 10.1016/j.crfs.2023.100515

**Published:** 2023-05-13

**Authors:** Sewuese S. Okubanjo, Sam J. Brooke, Rob Ward, Nic Mostert, Simon M. Loveday, Aiqian Ye, Peter J. Wilde, Harjinder Singh, Mark Waterland

**Affiliations:** a Riddet Institute, Massey University, Private Bag 11222, Palmerston North, 4442, New Zealand; b Norwegian University of Science and Technology (NTNU), Department of Biotechnology and Food Science, Trondheim, Norway; c Institute of Fundamental Sciences, Massey University, Private Bag 11222, Palmerston North, 4472, New Zealand; d Singapore Institute of Food and Biotechnology Innovation (SIFBI), Agency for Science, Technology and Research (A*STAR), 138673, Singapore; e Quadram Institute Bioscience, Norwich Research Park, Norwich, Norfolk, NR4 7UA, UK; f White Rabbit Scientific Limited, Palmerston North, New Zealand

**Keywords:** Droplet-stabilized emulsion, β-carotene, Microfluidic channel, Confocal Raman microscopy

## Abstract

This study sought to explore the combined use of confocal Raman microscopy and microfluidic channels to probe the location and mobility of hydrophobic antioxidant (β-carotene) incorporated at the interface of food-grade droplet-stabilized emulsions (DSEs).

Microfluidic channels were used to isolate emulsion droplets for efficient investigation of antioxidant mobility. This approach proved more conclusive than fixing the sample in agarose, because a single layer of droplets could be obtained. Results also indicated that the migration of β-carotene incorporated in shell droplets of olive oil and trimyristin DSEs to core droplets was minimal and beta-carotene remained mostly localised at the interface even after 3 days of production.

This work demonstrates that microfluidic isolation of emulsion droplets combined with confocal Raman microscopy can give new insights into the spatial variation of chemical composition within emulsions.

This study revealed that the migration of β-carotene between shell and core was minimal and hence it may be possible to concurrently deliver two incompatible compounds by spatially segregating them between shell and core compartments of DSEs.

## Highlights

• Microfluidic channels enabled the isolation of emulsion droplets for efficient probing of β-carotene location and mobility.
• Combination of confocal Raman microscopy and microfluidic channels provides a new method for studying β-carotene location and mobility in emulsion systems.
• β-carotene remained mostly localised within shell droplets of droplet-stabilized emulsions (DSEs).

## Graphical abstract

**Figure:**
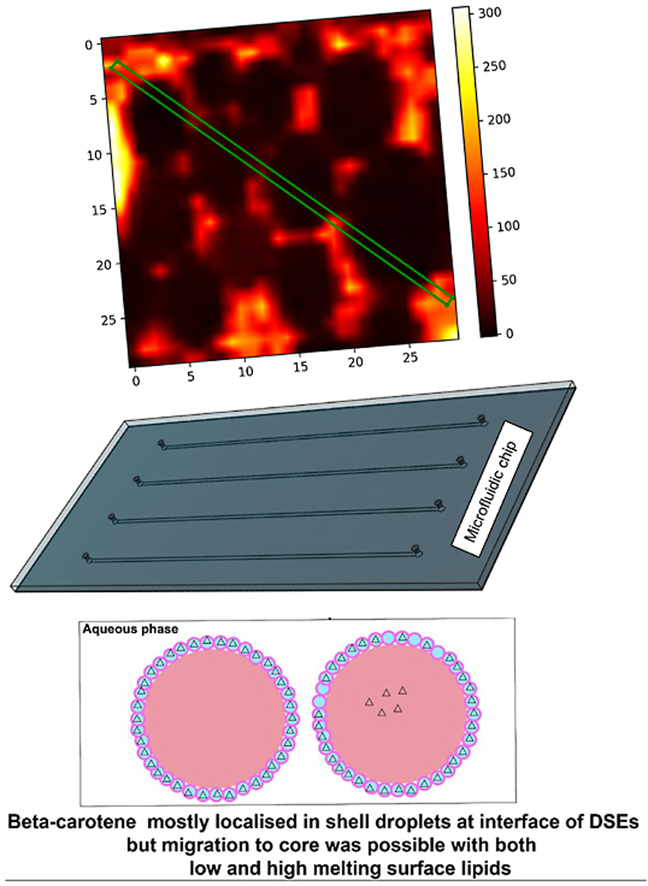

## 1 Introduction

The partitioning behaviour or pattern of an antioxidant incorporated within delivery systems such as emulsions influences the functional performance of antioxidants. Most partitioning studies to determine the distribution or precise location of antioxidants incorporated in emulsions involve separation of the phases and quantification of the antioxidant within each phase (Jacobsen et al., 1999; Pekkarinen et al., 1999; Stöckmann and Schwarz, 1999). The ability of antioxidants incorporated in oil-in-water emulsions to limit oxidation depends on their ability to counteract factors that promote oxidation and so location and mobility of antioxidants in oil-in-water emulsions are key factors influencing their antioxidant activity.

Droplet-stabilized emulsions (DSEs) consist of lipid droplets (the core) stabilized by sub-micron protein-stabilized lipid droplets (the shell) (Ye et al., 2013). DSEs effectively protects unsaturated lipids from oxidation (Okubanjo et al., 2019). The unique structure of DSEs presents the possibility of incorporating hydrophobic antioxidants in shell droplets located at the interface rather than in the interior of the core lipid. DSEs also have the potential to be used as a delivery system to concurrently deliver two different bioactive compounds by incorporating one in shell droplets and another in the core droplets. Our previous study showed that butylated hydroxyanisole (BHA) incorporated in olive and trimyristin shell droplets of DSEs limited oxidation of core safflower oil to a similar extent to when they were dispersed directly in core safflower oil of DSEs, and that location-based performance of BHA was dependent on concentration (Okubanjo et al., 2021).

This study sought to address the question of possible migration of hydrophobic antioxidants from shell droplets to core droplets of DSEs by testing whether antioxidants incorporated at the interface of droplet-stabilized emulsions (in shell droplets) remain localised at the interface or migrate over time into core droplets. This cannot be achieved using the conventional approach of separating the oil and water phases and requires a more advanced method to determine the partitioning in situ between two different oil phases.

In preliminary experiments, we explored the use of confocal Raman microscopy to examine the location and mobility of antioxidant by fixing the emulsions in agarose as is the practice with structure examination of emulsions with confocal laser scanning microscopy. However, fixing the emulsions in agarose made it very difficult to isolate single droplets. The problem was also compounded by the thickness of the sample. Therefore it appeared that Raman scattering was being collected from more than one droplet at once. To overcome this challenge, the possibility of isolating a single droplet was explored with the use of a microfluidic channel.

In this study the use of confocal Raman microscopy to monitor the location and mobility of hydrophobic antioxidant incorporated in shell droplets of DSEs was investigated using microfluidic channels to isolate the droplets.

## 2 Materials and methods

### 2.1 Materials

Milk Protein concentrate (MPC-70) was kindly donated by Fonterra Cooperative Ltd, Auckland, NZ. Trimyristin (Dynasan 114) was kindly donated by IOI Oleo GmbH, Germany. High linoleic acid (72.54% of total fatty acids) refined safflower oil was purchased from Pure Nature Ingredients, Auckland New Zealand. Olive oil (low acidity) and β-carotene were purchased from Sigma-Aldrich.

### 2.2 Emulsion preparation

Lipid phases were prepared by stirring and heating β-carotene into the surface or core lipid for 15 min at 70 °C. The total amount of β-carotene was the same for emulsions with β-carotene in the core lipid (core-loaded β-carotene DSE) and emulsions with β-carotene in the surface lipid (surface-loaded β-carotene DSE). Table 1 summarizes and shows a description of the emulsions processed for the study and final amounts of β-carotene in emulsions. Droplet-stabilized emulsions with β-carotene incorporated in shell or core droplets were prepared by a two-step process as previously described by Okubanjo, Loveday, Ye, Wilde and Singh (Okubanjo et al., 2019) with slight modifications.

**Table 1:** Average particle size of β-carotene loaded droplet-stabilized emulsions.

Sample codes	Description	Surface lipid	Beta-carotene Location & amount (ppm)	Surface-weighted mean diameter, d_3,2_ (μm)	Volume weighted mean diameter, d_4,3_ (μm)
NOS	DSE with β-carotene-in-shell	Olive oil	Shell (100 ppm)	7.52 ± 0.2^a^	13.48 ± 0.1^a^
NOC	DSE with β-carotene -in-core- **β-carotene amount matched with NOS**	Olive oil	Core (100 ppm)	8.00 ± 0.4^a^	13.76 ± 0.3^a^
NTS	DSE with β-carotene -in-shell	Trimyristin	Shell (100 ppm)	7.33 ± 0.2^a^	14.31 ± 0.6^a^
NTC	DSE with β-carotene -in-core- **β-carotene amount matched with NTS**	Trimyristin	Core (100 ppm)	6.77 ± 0.1^a^	12.47 ± 0.1^a^

Firstly, solutions consisting of 5% (w/w) milk protein concentrate (MPC) were made by stirring MPC powder (5 g) into Milli-Q water (95 g) at room temperature for 1 h. The MPC solution was heated to 65 °C and appropriate quantities of surface lipid phase were added. The mixtures were held at 65 °C for 3–5 min and coarse emulsions made using a high-speed mixer (Labserv, D-130) at 22000 rpm for 3 min. The coarse emulsion was passed through a two-stage homogenizer (Homolab2, FBF Italia) at a first stage pressure of 400 bar and second stage pressure of 50 bar to produce a fine emulsion which is referred to as ‘shell emulsion’. In the second step, the shell emulsion was added to appropriate quantities of PUFA-rich lipid (safflower oil), which is referred to as ‘core lipid,’ and potassium phosphate buffer (10 mM K_2_HPO_4_ and KH_2_PO_4_, pH 6.8–7.0), i.e. 10% w/w shell droplets, 20% w/w core lipid and 70% w/w buffer. To form droplet-stabilized emulsions, the mixture was heated to 65 °C and processed with a high-speed mixer (D-130, Labserv) at 22000 rpm for 5 min.

Composition-matched control emulsions were also processed according to the method described by Okubanjo, Loveday, Ye, Wilde and Singh (Okubanjo et al., 2019) Shell emulsions with or without beta-carotene were added to appropriate quantities of protein-stabilized conventional oil-in-water emulsion which was processed by mixing appropriate quantities of 0.4% w/v MPC solution and safflower oil with a high-speed mixer.

### 2.3 Particle size characterization

The surface area mean (Sauter mean diameter, d_3,2_) and volume weighted mean diameter (d_4,3_) of emulsions were measured using a static light scattering instrument (Mastersizer, 2000; Malvern instruments, Worcestershire, UK). The emulsions were dispersed in reverse osmosis-filtered water and the ratio of refractive index of emulsion droplets (1.47) to the aqueous medium (1.33) was 1.105. The measurements were taken in triplicate.

### 2.4 Microfluidic channel

**Fig. 1:**
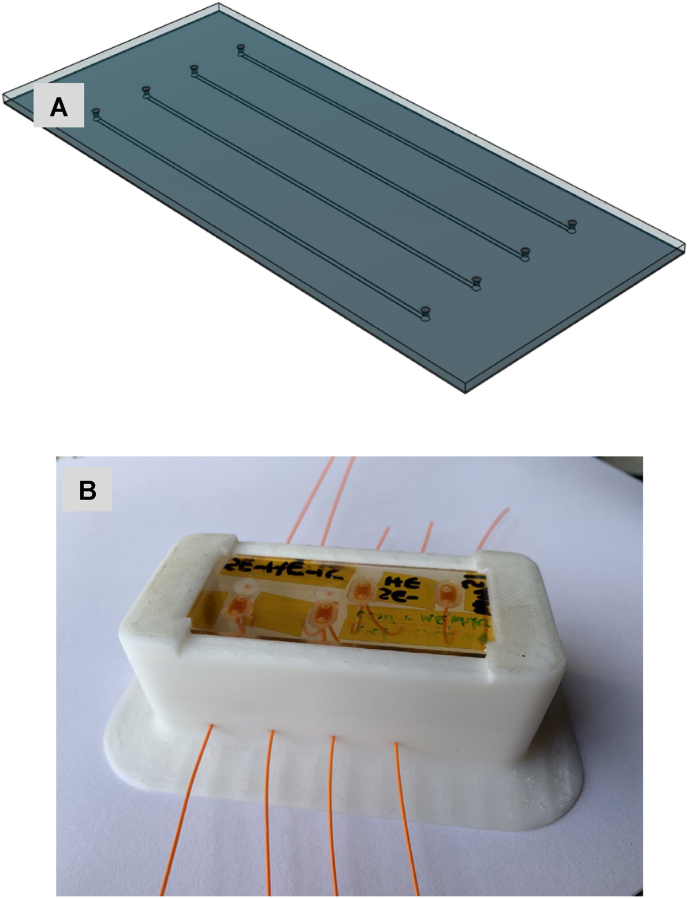
CAD drawing (A) and photo (B) of mircofluidic chip.

The microfluidic chip was manufactured from borosilicate glass. The channel dimensions are 20 mm (length) x 500 μm (width) x 50 μm (depth) and were created using chemical etching. Fluidic portholes at each end of the channel enabled connection of 1/32 inch (0.8 mm) tubings (Fig. 1A and B). Emulsion was injected into the microfluidic channel using a precision 50 μL Teflon tipped glass syringe.

### 2.5 Confocal Raman spectroscopy

The location and mobility of antioxidant incorporated in droplet-stabilized emulsion was probed using a confocal WITec alpha300 RA Raman microscope. Raman data was collected on the same microscope with a 532 nm source between 3 mW and 15 mW at the sample. 50 μL of emulsion was loaded into a 15 μm deep microfluidic channel chip. The loaded microfluidic channel chip was mounted to a fixed point on the electronic XYZ three-axis stage, where the microfluidic channel position and focal depth were recorded for ease of alignment between samples. The emulsions were brought into focus and then 1D, 2D or 3D scans in this region were performed. For beam-sensitive 1D scans, lower power was used, and the laser was blocked immediately before and after scans to minimize laser-induced antioxidant degradation during alignment on the droplets. The location of antioxidant was probed after 3 days production of DSEs.

The detection limit for this technique is dependent on the scattering activity of each molecule in the sample, wavelength, and experimental setup.

### 2.6 Raman data analysis

Raman data was exported from WITec project 2.10 software via CytoSpec (version 2.00.05) hyperspectral imaging software. The data was exported from CytoSpec into a compatible format and Raman spectra plots obtained using Python 3.0 software. To plot Raman spectra corresponding to selected emulsion droplets, image maps were plotted, and a line drawn or scanned across droplets using x, y coordinates from the mapped image. Raman spectra corresponding to the points along the drawn lines were obtained and Raman intensities of components of interest along the scanned area (line scan) plotted.

## 3 Results

### 3.1 Particle size of beta-carotene loaded droplet-stabilized emulsions

The average (*d*_*3,2*_) and volume (*d*_*4,3*_) weighted mean diameters of olive DSEs with beta-carotene-in-shell and trimyristin DSEs with β-carotene-in-shell were similar (Table 1). The average (*d*_*3,2*_) and volume (*d*_*4,3*_) weighted mean diameters of DSE with β-carotene-in-shell and in core were also similar.

### 3.2 Confocal Raman microscopy (microfluidic channel)

**Fig. 2:**
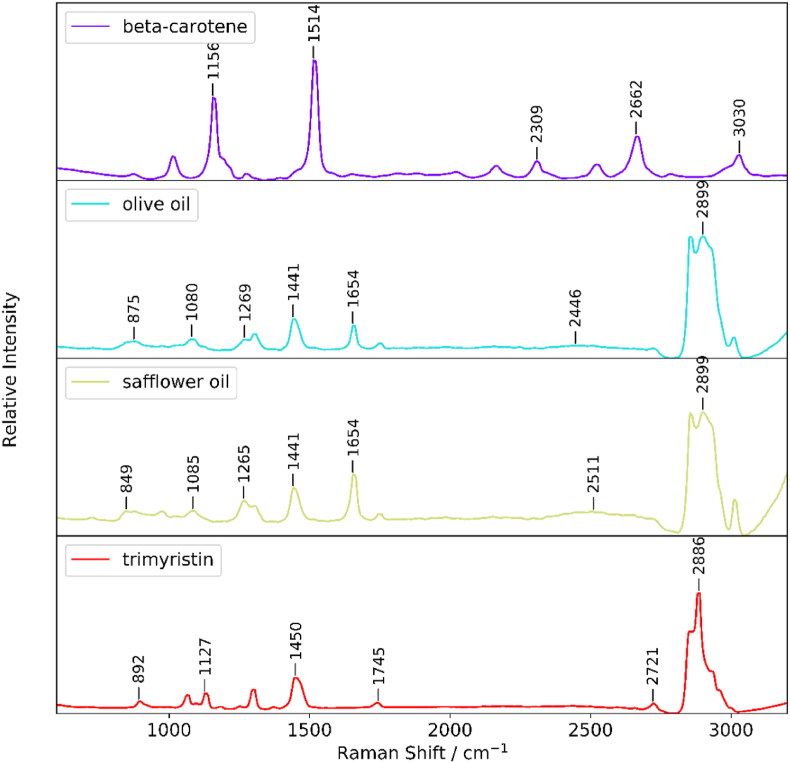
Raman spectra of β-carotene, olive oil, safflower oil, and trimyristin.

β -carotene has two intense Raman bands at 1156 cm^−1^ and 1514 cm^−1^, whereas olive oil, safflower oil and trimyristin have intense bands from their C–H groups at about 2886–2899 cm^−1^ (Fig. 2). Olive oil and safflower oil have slightly intense bands at 1654 cm^−1^.

**Fig. 3:**
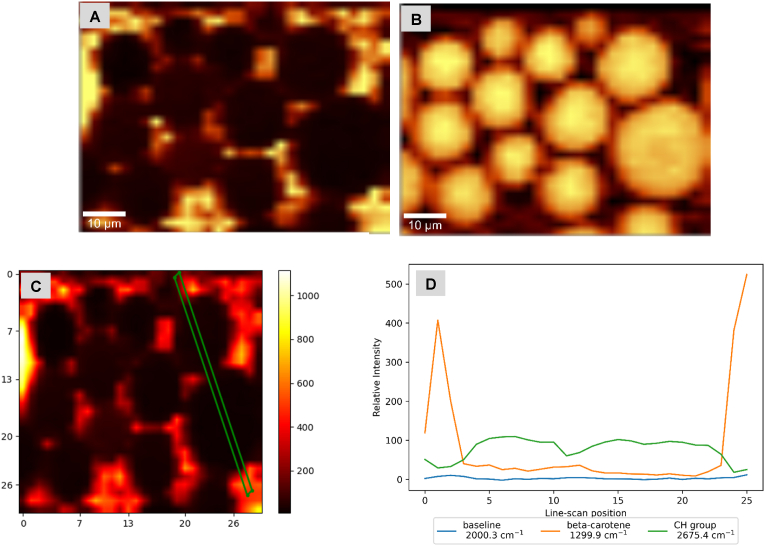
Confocal Raman microscopy images and intensity profile of Olive oil DSEs with β-carotene-in-shell droplets- A & B = β-carotene, and CH band Raman channels respectively; C= Line scan location across droplets (X and Y scales are in microns); D = Intensity profiles for selected Raman channels across line scan shown in C.

**Fig. 4:**
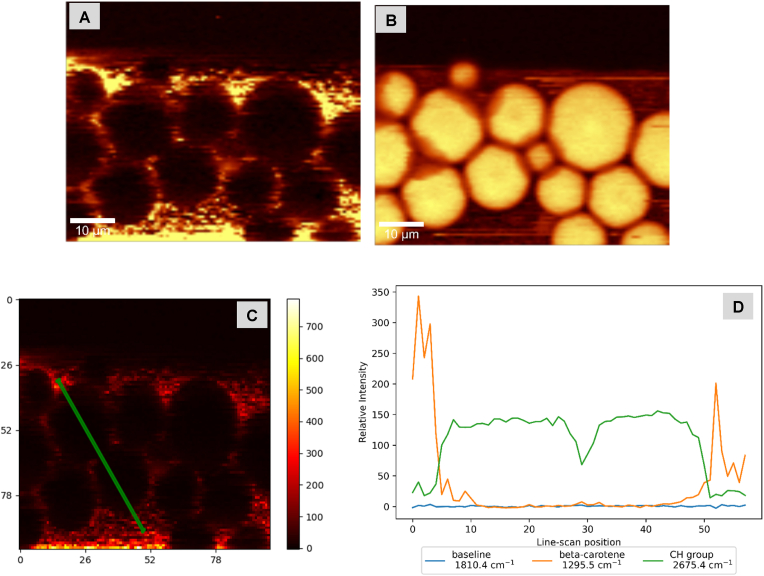
Confocal Raman microscopy images and intensity profile of trimyristin DSEs with β-carotene-in-shell droplets- A & B = β-carotene, and CH band Raman channels respectively; C= Line scan location across droplets (X and Y scales are in microns); D = Intensity profiles for selected Raman channels across line scan shown in C.

**Fig. 5:**
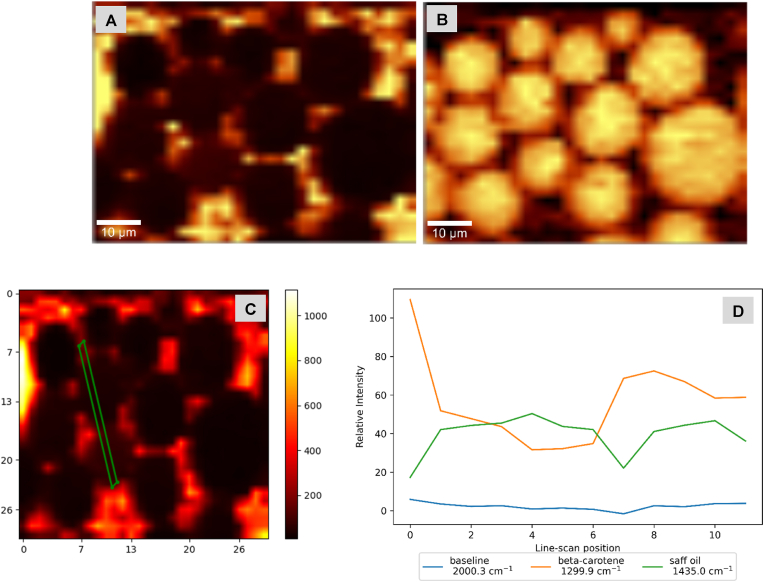
Confocal Raman microscopy images and intensity profile of olive DSEs with β-carotene-in-shell droplets showing evidence of possible migration to core **–** A & B = β-carotene and safflower oil Raman channels respectively; C= Line scan location across droplets (X and Y scales are in microns); D = Intensity profiles for selected Raman channels across line scan shown in C.

**Fig. 6:**
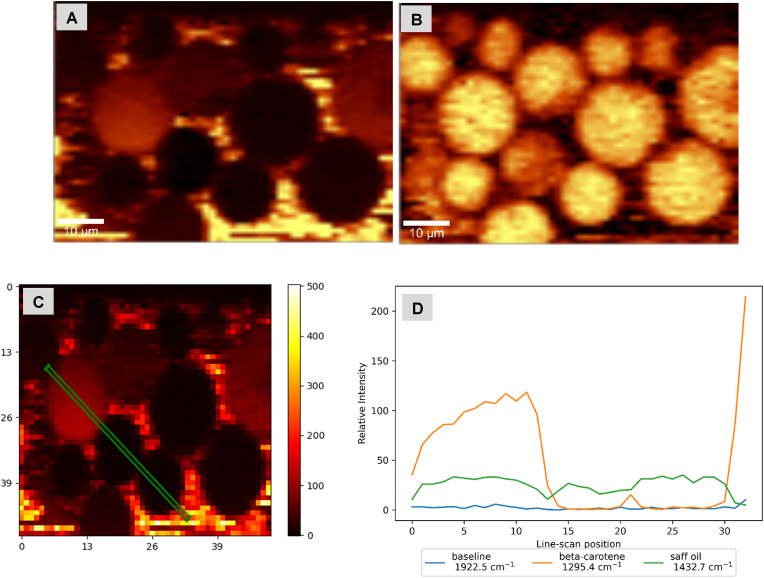
Confocal Raman microscopy images and intensity profile of trimyristin DSEs with β-carotene-in-shell droplets showing evidence of possible migration to core- A & B = β-carotene and safflower oil Raman channels respectively; C= Line scan location across droplets (X and Y scales are in microns); D = Intensity profiles for selected Raman channels across line scan shown in C.

For β-carotene-in-shell DSEs made with olive oil (Fig. 3) or trimyrstin (Fig. 4), two confocal Raman ‘channels’ (channels refers to a specific frequency or band of frequencies) are shown: β-carotene, and CH band channels. Confocal Raman images and Raman spectral plots of olive oil and trimyristin control emulsions, and DSEs with β-carotene-in-core are shown in supplementary material (see supplementary information-Figs. 5,6,7&8). Control emulsions consisted of β-carotene loaded shell droplets gently stirred into protein-stabilized safflower oil droplets, shell droplets were dispersed in the surrounding phase with some spontaneous adsorption. Scanning from shell droplets surrounding the interface of core droplet and across the droplet (see supplementary information-Figs. 5 and 7) showed changes in the Raman intensity of β-carotene.

The Raman intensity profile across olive DSEs droplets with β-carotene-in-shell droplets (Fig. 3C) showed high β-carotene intensities and low CH band intensities at line scan positions (∼0–3 and 23–25) corresponding to the interfaces of safflower oil droplets (Fig. 3D), while low β-carotene intensities and high CH band intensities were observed at line scan positions (4–23) corresponding to core safflower oil droplets (Fig. 3D). The reverse trend was observed with intensity profiles of olive DSEs droplets with β-carotene-in-core safflower oil droplets (see supplementary information-Fig. 6).

Trimyristin DSEs (Fig. 4) gave similar results: high β-carotene and low CH band intensities at line scan positions (∼0–5 and 48–50) corresponding to the droplet interfaces, and low β-carotene and high CH band intensities at line scan positions (∼12–45) corresponding to the core (Fig. 4D). Again, the β-carotene-in-core safflower oil droplets (see supplementary information-Fig. 8) gave the reverse trend.

Fig. 5 shows confocal Raman images and intensity profile plots of olive DSEs with β-carotene incorporated in shell droplets. Two confocal Raman ‘channels’ are shown: β-carotene, and safflower oil channels. Raman intensity profile shows high β-carotene intensities at line scan positions (∼7–10) corresponding to core safflower oil droplets (Fig. 5 D). In the trimyristin oil DSEs (Fig. 6), high β -carotene intensities at line scan positions (∼1–12) corresponding to core safflower oil droplets was also observed (Fig. 6D). Raman spectra at line scan positions corresponding to the interior of the droplet in both the olive and trimyristin DSEs indicated the presence of β-carotene in the core of a few droplets.

Photo-bleaching of β-carotene provides an explanation for why some β-carotene-in-core droplets did not appear to have β-carotene (see supplementary information-Fig. 6A&D). The β-carotene channels from confocal Raman images of DSEs with β-carotene-in-core safflower oil droplets analysed at high power exposure (15 mW) indicate some photo-bleaching occurred while scanning at high power (see supplementary information-Fig. 9).

## 4 Discussion

β-carotene was chosen for this study because it exhibits very strong intrinsic Raman scattering, characterised by the Raman scattering cross-section. Raman intensities of β-carotene and other analysed components (safflower oil, CH band) differ, so Raman scattering intensities are not directly indicative of concentration and can be interpreted only in relative terms.

Use of a microfluidic channel to isolate a single layer of droplets made it possible to overcome the challenge experienced with collecting Raman data when DSEs were fixed in agarose (see supplementary information-Fig. 2, Fig. 3, Fig. 4). Based on the results obtained with the microfluidic channel, β-carotene incorporated in shell droplets of DSEs remained mostly localised within the shell droplets but, noticeable β-carotene signals obtained within the core of some safflower oil droplets confirm limited migration of β-carotene from shell to core.

The emulsions were analysed after 3 days of production and all samples were analysed within 3–5 days of production, so it is difficult to evaluate at what point the observed β-carotene exchange from shell to core occurred. However, based on the Raman data of the control emulsions where no high-speed mixing was done but rather the shell droplets containing β-carotene was gently stirred in, there were no β-carotene signals in the core safflower oil indicating there was no migration or diffusion from the gently stirred-in shell to core. This suggests that migration occurred during high-speed mixing.

The absence of β-carotene signals in core safflower oil droplets of control emulsions (see supplementary information-Figs. 5 and 7) indicate that β -carotene exchange from shell to core in DSEs may have occurred during adsorption of shell droplets to core droplets in the final mixing step of DSE formation. However, considering the time scale required for shell droplets to adsorb to the interface of core droplets is very short, it is unlikely that β-carotene transfer occurred by diffusion across the interface. β-carotene transfer from shell to core may have occurred through occasional coalescence between shell and core droplets. β-carotene transfer from shell to core was minimal so it implies that the emulsions are relatively stable to coalescence, or the level of coalescence is so low that β -carotene concentrations are small in core droplets.

Only a small amount of emulsion was introduced into the microfluidic channel for analysis, so results may not be fully representative of the whole sample, but consistency between different emulsions suggests that small field of view did not compromise representativeness. The emulsions were analysed 3 days after preparation due to logistical limitations (different locations for sample preparation and analysis), so it is quite difficult to conclude that the β-carotene exchange in DSEs occurred during shell droplet adsorption in the final mixing step.

Overlaps in Raman signals of the lipids (olive, safflower and trimyristin-Fig. 2) and inability to identify unique intense signals from trimyristin and olive oil made it difficult to evaluate whether β-carotene exchange from shell to core was due to surface lipid exchange to core droplets, or due to β-carotene diffusion from shell droplets across the protein interface to the core.

Results from this study do not provide a quantitative measure to enable comparative analysis between low (olive) and high (trimyristin) melting DSEs in terms of levels of β-carotene exchange from shell to core, and if the exchange was slower with high melting shell droplets however, in previous studies (Okubanjo et al., 2019, 2021), we reported and discussed in detail the impact of DSEs interfacial structure, interfacial composition and droplet size on the oxidative stability and antioxidant performance of olive and trimyristin DSEs and based on our previous observations, it is possible that the differences in shell droplet lipid state (i.e. liquid vs solid) between olive and trimyristin DSEs will have an effect on the levels of β-carotene exchange from shell to core.

Another challenge observed with the confocal Raman study was that at high power exposure, β-carotene was susceptible to photo-bleaching and so it was difficult to determine the extent of β-carotene exchange. Overall, observation of most DSEs droplets at low power exposure showed that β-carotene remained localised within shell droplets.

Label-free imaging of bioactive molecules in food products can also be achieved with more sophisticated Raman techniques such as coherent anti-stokes Raman scattering (Day et al., 2010) and stimulated Raman scattering Raman microscopy (Roeffaers et al., 2011) however, one of the advantages of the confocal Raman microscopy and microfluidic channel method is that it does not require any complex set-up and analysis can be achieved with widely available lab-scale Confocal Raman microscope.

In conclusion, results from this study shows that microfluidic channels can be used to isolate DSE droplets to enable efficient location probing and diffusion or mobility studies of antioxidants or other components incorporated within. The results also indicate that antioxidants or bioactives incorporated in shell droplets of DSEs may remain mostly localised within the shell droplets, but limited migration may occur either due to high-speed mixing of shell droplets and core droplets or overtime.

This study demonstrated a new method that can be used to study antioxidant location and mobility in emulsion systems without separating emulsion phases and has also provided critical information about the possible fate of antioxidants incorporated in shell droplets of DSEs. The method can also be used to examine the location of compounds in other complex food systems.

## CRediT authorship contribution statement

**Sewuese S. Okubanjo:** Conceptualization, Data curation, Formal analysis, Investigation, Methodology, Writing – original draft. **Sam J. Brooke:** Methodology, Validation. **Rob Ward:** Methodology, Resources. **Nic Mostert:** Software. **Simon M. Loveday:** Supervision, Conceptualization, Writing – review & editing, Funding acquisition. **Aiqian Ye:** Supervision, Conceptualization. **Peter J. Wilde:** Supervision, Conceptualization, Writing – review & editing. **Harjinder Singh:** Supervision, Conceptualization, Writing – review & editing, Funding acquisition. **Mark Waterland:** Conceptualization, Data curation, Software, Methodology, Resources, Validation, Writing – review & editing.

## Declaration of competing interest

The authors declare that they have no known competing financial interests or personal relationships that could have appeared to influence the work reported in this paper.

## Data Availability

Data will be made available on request.
