# Supplementary material for: The use of confocal Raman microscopy and microfluidic channels to monitor the location and mobility of β-carotene incorporated in droplet-stabilized oil-in-water emulsions
Source: Curr Res Food Sci. 2023 May 13;6:100515. doi: 10.1016/j.crfs.2023.100515 (PMC10205456; doi:10.1016/j.crfs.2023.100515)
Supplement: Multimedia component 1 [file mmc1.pdf]

Supplementary Information

Confocal Raman Microscopy (microscope slides)

Figure 1 shows Raman spectra of β-carotene loaded DSEs. The red trace shows the intensity profile of the carotene band from the Raman spectrum (shaded red) along the line (also shown in red) in the optical image. A unique olive oil band was identified at ~ 1650 cm^{-1}. The band at ~ 2900 cm^{-1} is due to overlapping contributions from various C-H vibrational modes of the emulsion components. The green and blue traces show the intensity profile of olive oil and CH bands respectively. The intensity profile correlates to the red line scan in the optical images shown in Figure 2, Figure 3, and Figure 4.

Figure 2 shows bright field reflectance optical images and Raman spectra corresponding to the line scanned region of DSE without β-carotene droplets in the optical image. Figure 3 and Figure 4 also shows the optical images and Raman spectra corresponding to the line scanned regions of DSEs with β-carotene incorporated in shell droplets and β-carotene incorporated in core droplets respectively.

As expected, there were no spikes in Raman intensity of β-carotene as DSE droplets without β-carotene were scanned because there was no β-carotene incorporated but changes in intensities of the CH band was observed (Figure 2b & d).

As DSE droplets with β-carotene-in-shell droplets were scanned (Figure 3a), increases or spikes in Raman intensity of β-carotene at the interfaces of DSE droplets were recorded and gradual decreases recorded as the scan continued across the core droplets (Figure 3b & d). The reverse case was observed with

DSE droplets with β-carotene-in-core droplets whereby spikes in Raman intensity of β-carotene (Figure 4b & d) were recorded while scanning through the core droplets (Figure 4a).

However, fixing the emulsions in agarose made it very difficult to isolate single droplets. The problem was compounded by the thickness of the sample as well, therefore it is likely that Raman scattering was being collected from more than one droplet at once. To overcome this challenge, the possibility of isolating a single droplet was explored with the use of a microfluidic channel.

Confocal Raman microsocpy results from the DSEs fixed in agarose indicated that β-carotene incorporated in shell droplets of DSEs remained localised within the droplets but looking at the Raman spectra there appeared to be strong β-carotene signals in scanned areas corresponding to core safflower oil droplets and the inablity to isolate droplets made it difficult to evaluate possible β-carotene migration.

Confocal Raman Microscopy (Microfluidic channels)

Microfluidic channels were used to overcome the challenge highlighted above.

Figure 5 and Figure 6 show confocal Raman images and Raman spectral plots of olive oil control emulsions, and DSEs with β-carotene-in-core respectively while, Figure 7and Figure 8 shows that of trimyristin (processed above 56°C) control emulsions, and DSEs with β-carotene-in-core droplets respectively.

Raman intensity profile of olive control emulsions (Figure 5E) showed high intensities of β-carotene at line scan positions (~ 0-5, 20-30 & 40-50) corresponding to interfacial regions with olive oil shell droplets containing β-carotene while very low β-carotene intensities were obtained at line scan positions (10-20, 30-40) corresponding to core safflower oil droplets. The same trend was observed with trimyristin control emulsions (Figure 7E) which showed high intensities of β-carotene at line scan positions (~ 0-3, 17-21 & 36-40) corresponding to interfacial regions with trimyristin shell droplets containing β-carotene while very low β-carotene intensities were obtained at line scan positions (~ 3-16 & 23-35) corresponding to core safflower oil droplets.

Raman intensity profile across olive DSEs droplets with β-carotene-in-core afflower oil droplets (Figure 6E) showed high β-carotene and high CH band intensities at line scan positions (~ 2-14) corresponding to core safflower oil droplets. Low β-carotene and low CH band intensities were also observed at line scan positions (~ 0-1) correspondng to the interfaces of core safflower oil droplets composed of shell droplets without β-carotene.

Raman intensity profile of trimyristin DSEs with β-carotene-in-core safflower oil droplets (Figure 8E) showed high β-carotene and high CH band intensities at line scan positions (~ 3-11 & 15-20) corresponding to core safflower oil droplets, and low β-carotene intensities were observed at line scan positions (~ 10-12) correspondng to the interfaces of core safflower oil droplets composed of shell droplets without β-carotene.

Figure 9 shows confocal Raman images of DSEs with β-carotene-in-core safflower oil droplets analysed at high power exposure (15 mW). The β-carotene channels indicate some photo-bleaching occured while scanning at high power.

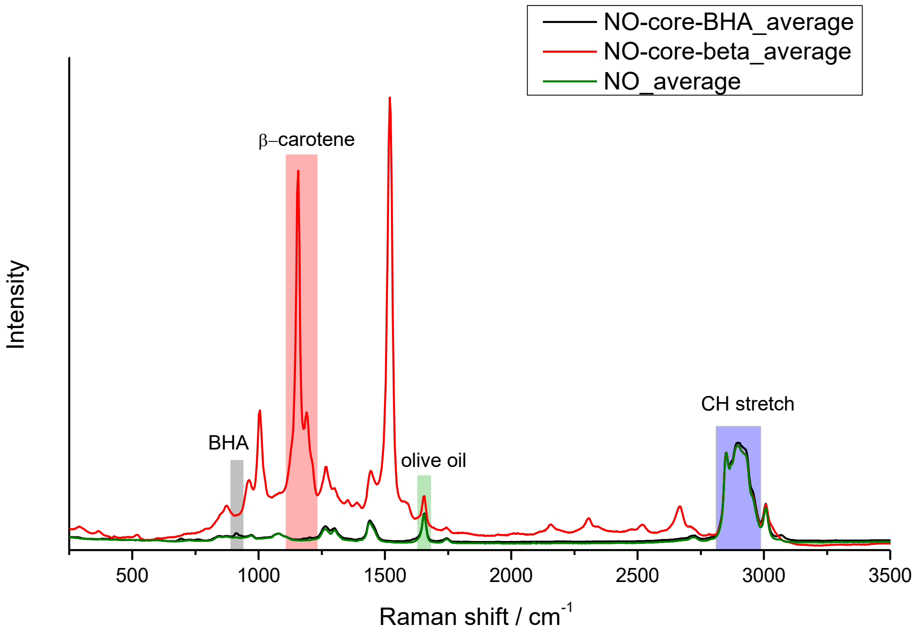

Figure 1: Raman spectra of DSEs with and without antioxidants (BHA & β-carotene)

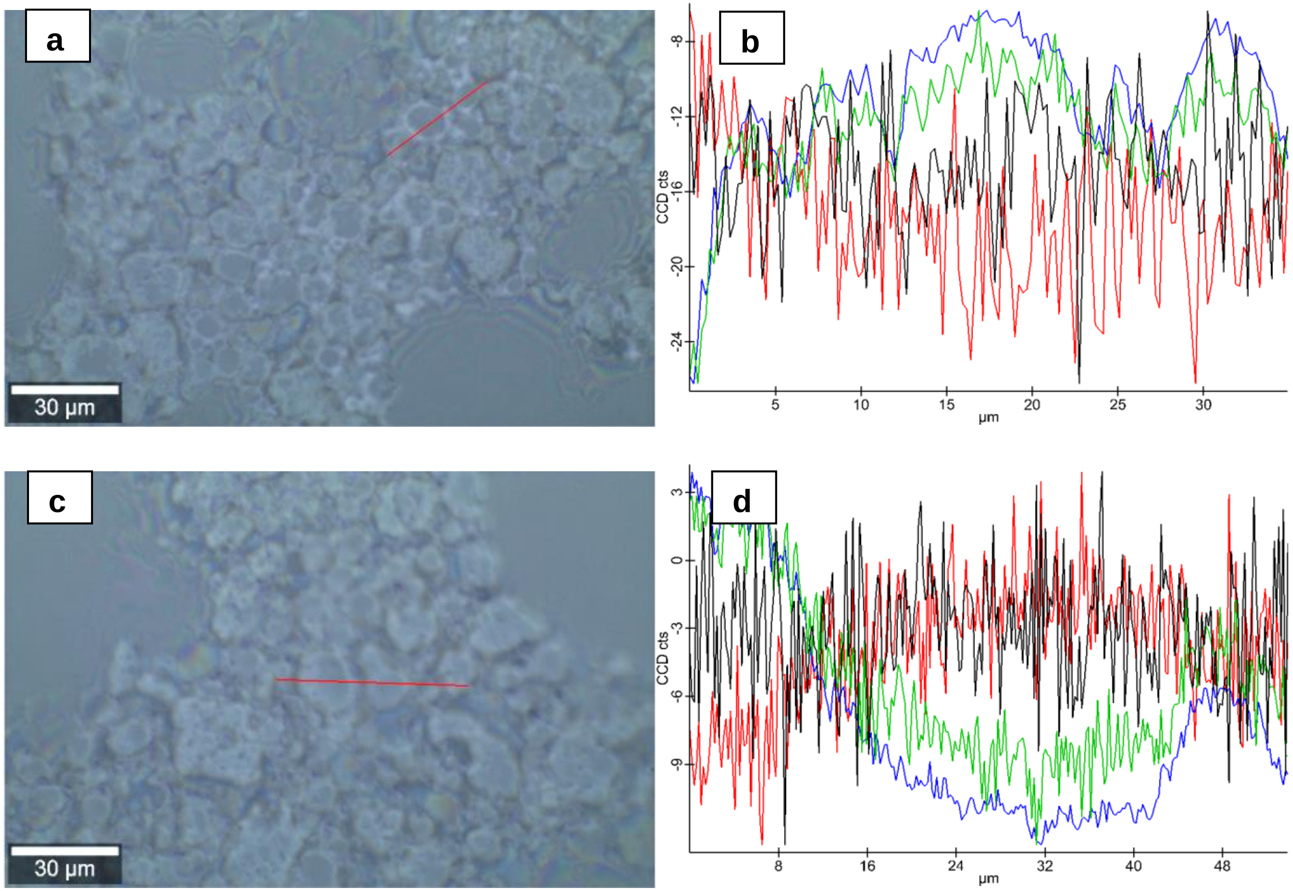

Figure 2: Olive oil DSE (without B-carotene) with 150 points (top) & 265 points (bottom) (a & c) bright field reflectance mode images, (b & d) peak integral distribution (red trace= β-carotene; blue trace= CH band; green trace= olive oil).

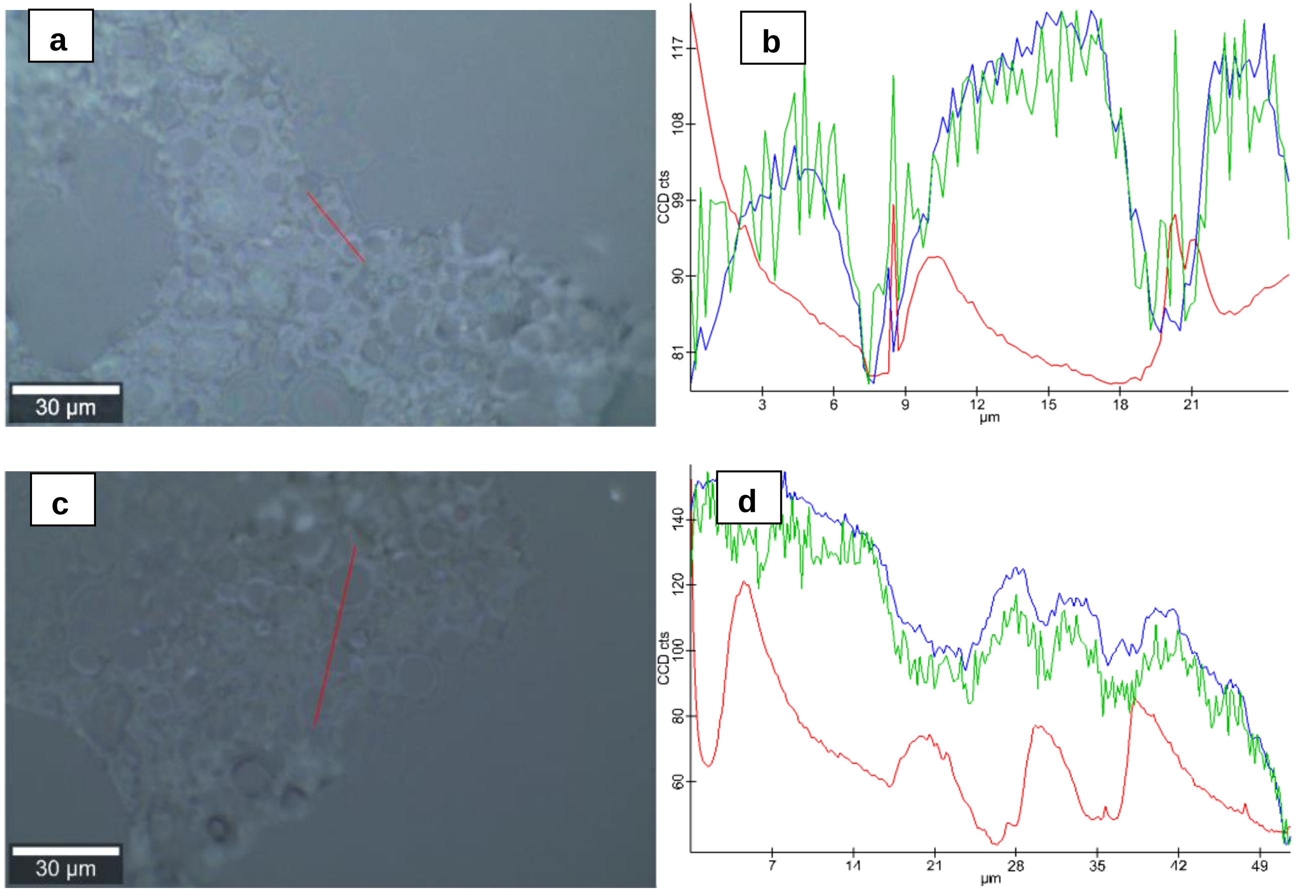

Figure 3: Olive DSE (beta-in-shell) with 122 points (top); & 250 points (bottom). (a & c) bright field reflectance mode image, (b & d) peak integral distribution (red trace= β-carotene; blue trace= CH band; green trace= olive oil).

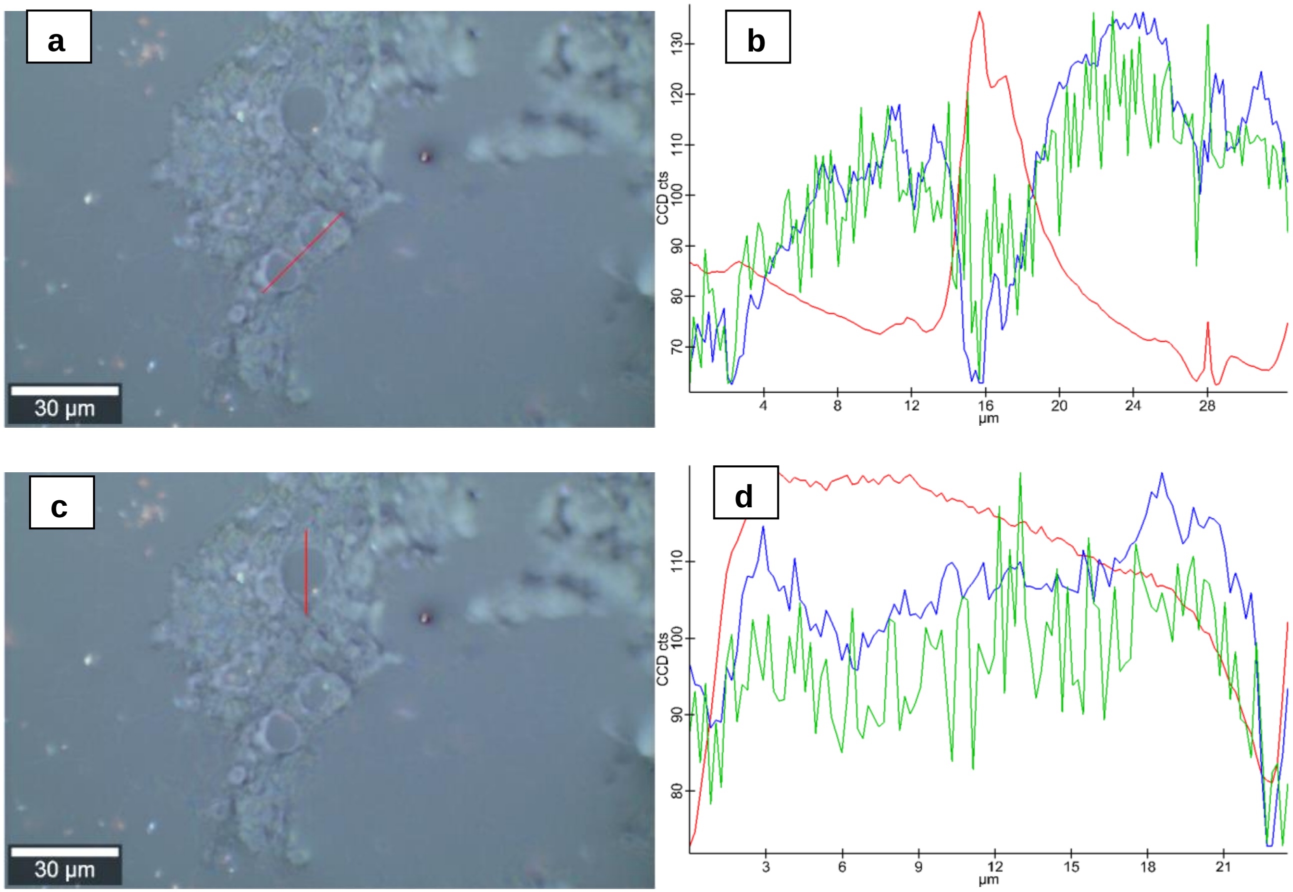

Figure 4: *Olive DSE (beta-in-core) with 158 points (top) & 115 points (bottom). (a & c) bright field reflectance mode image, (b & d) peak integral distribution (red trace= β-carotene; blue trace= CH band; green trace= olive oil).*

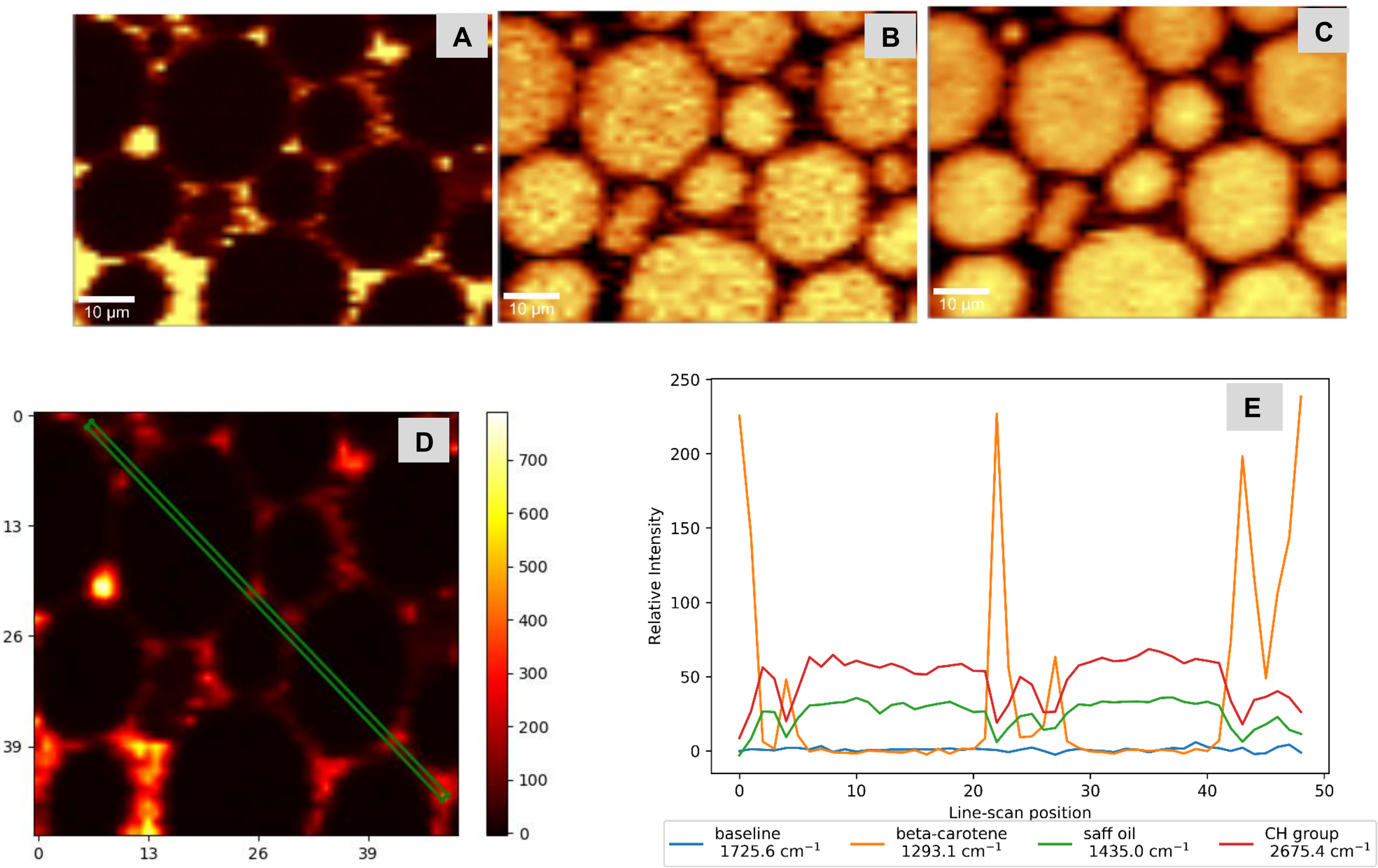

Figure 5: Confocal Raman microscopy images and intensity profile of Olive oil control emulsions (shell gently stirred-in) - A, B, & C= β-carotene, safflower oil and CH band Raman channels respectively; D= Line scan location across droplets; E= Intensity profiles for selected Raman channels across line scan shown in D.

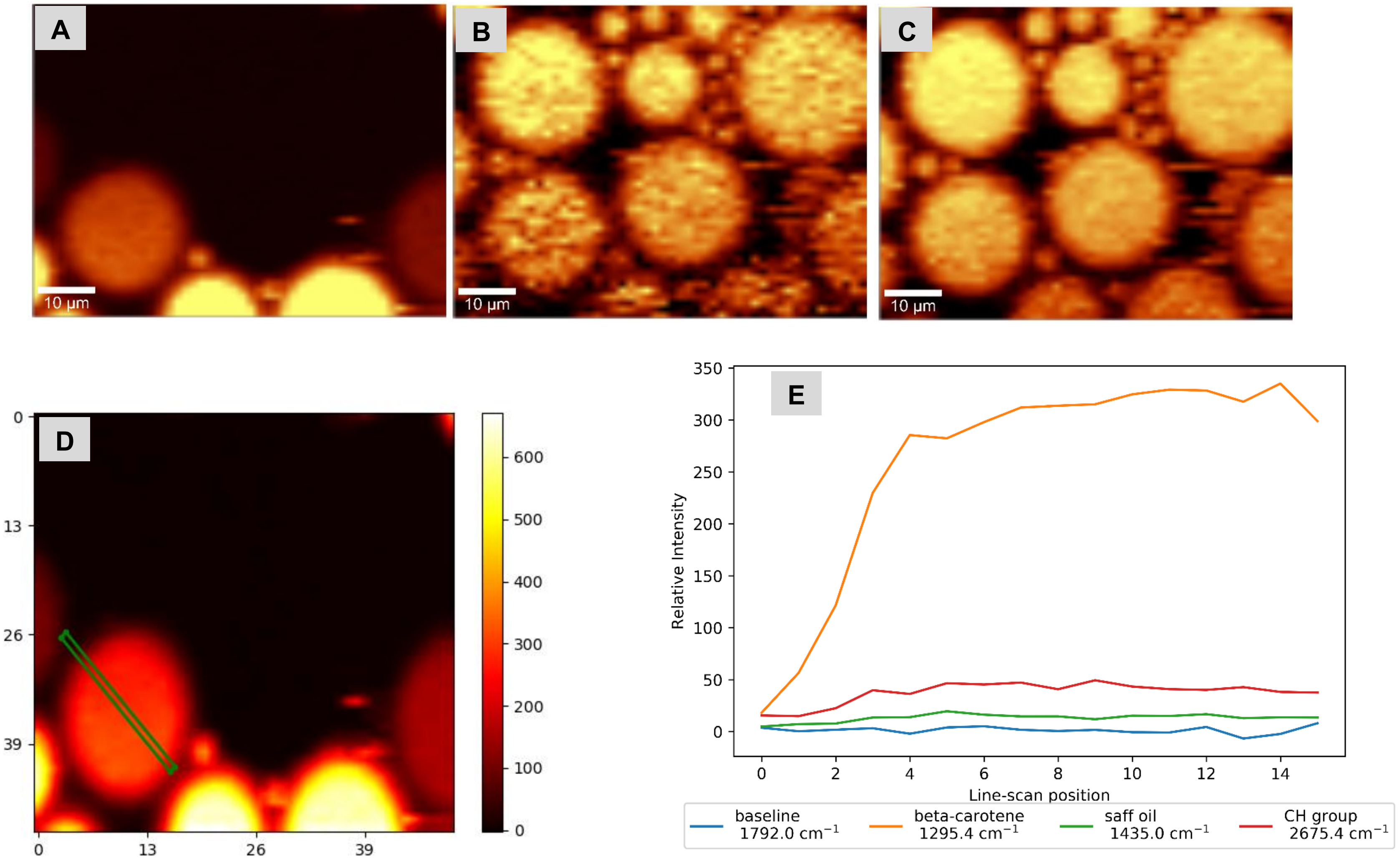

80 *Figure 6: Confocal Raman microscopy images and intensity profile of olive oil DSEs with β-carotene-in-core droplets- A, B, & C= β-carotene, safflower oil and CH band Raman channels respectively; D= Line scan location across droplets; E= Intensity profiles for selected Raman channels across line scan shown in D.*

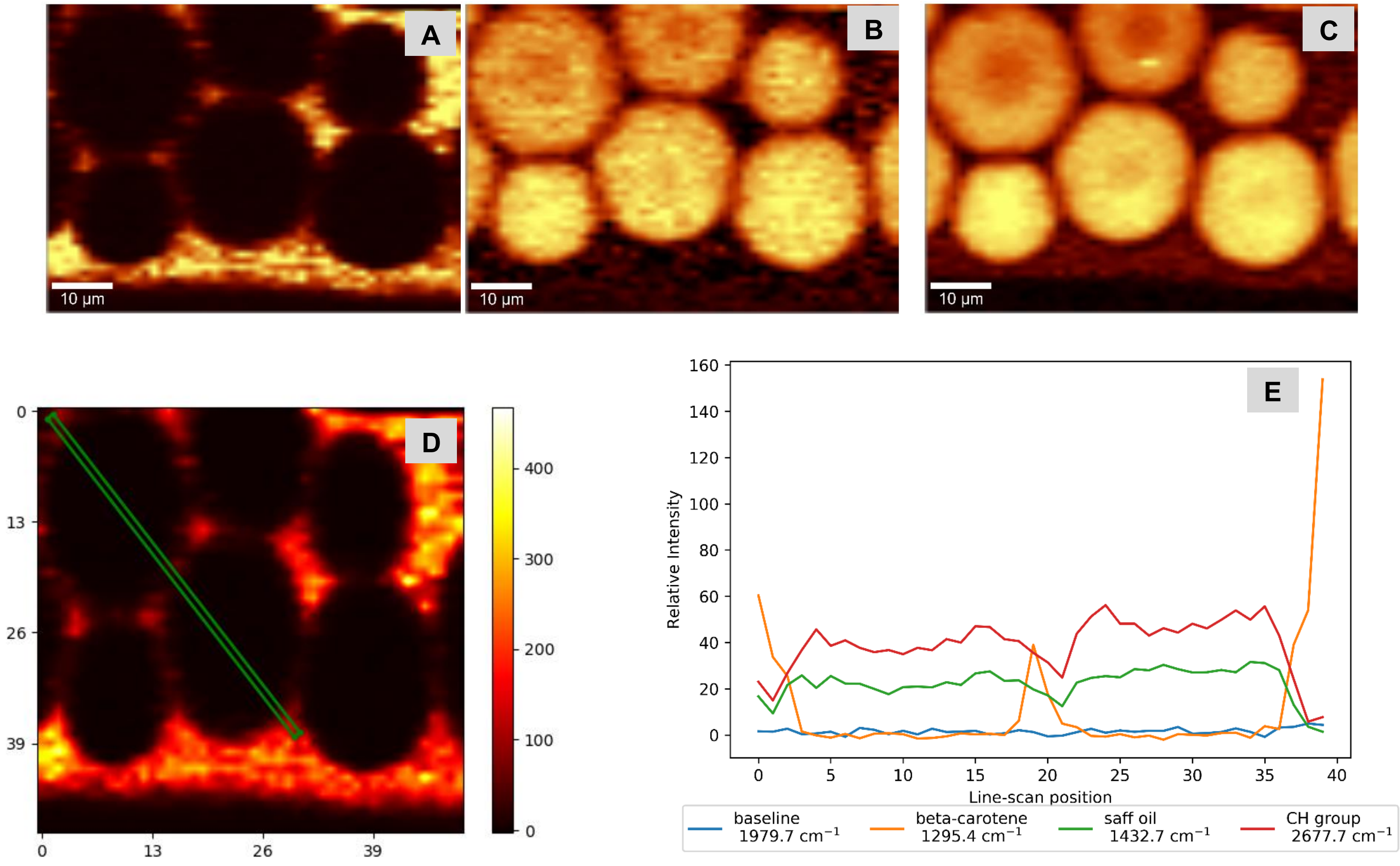

Figure 7: Confocal Raman microscopy images and intensity profile of trimyristin control emulsion (shell gently stirred-in) - A, B, & C= β-carotene, safflower oil and CH band Raman channels respectively; D= Line scan location across droplets; E= Intensity profiles for selected Raman channels across line scan shown in D.

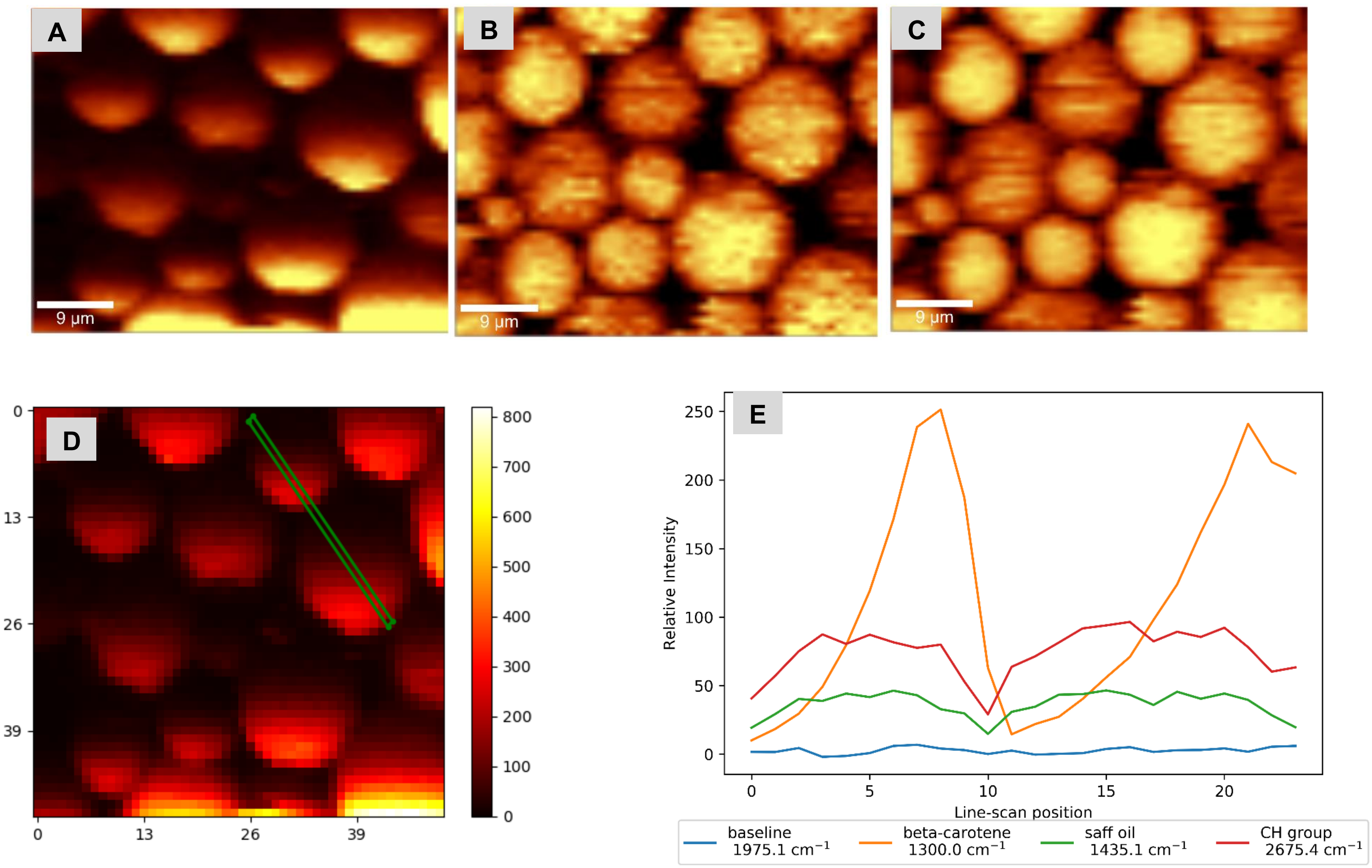

Figure 8: Confocal Raman microscopy images and intensity profile of trimyristin DSEs with β-carotene-in-core droplets- A, B, & C= β-carotene, safflower oil and CH band Raman channels respectively; D= Line scan location across droplets; E= Intensity profiles for selected Raman channels across line scan shown in D.

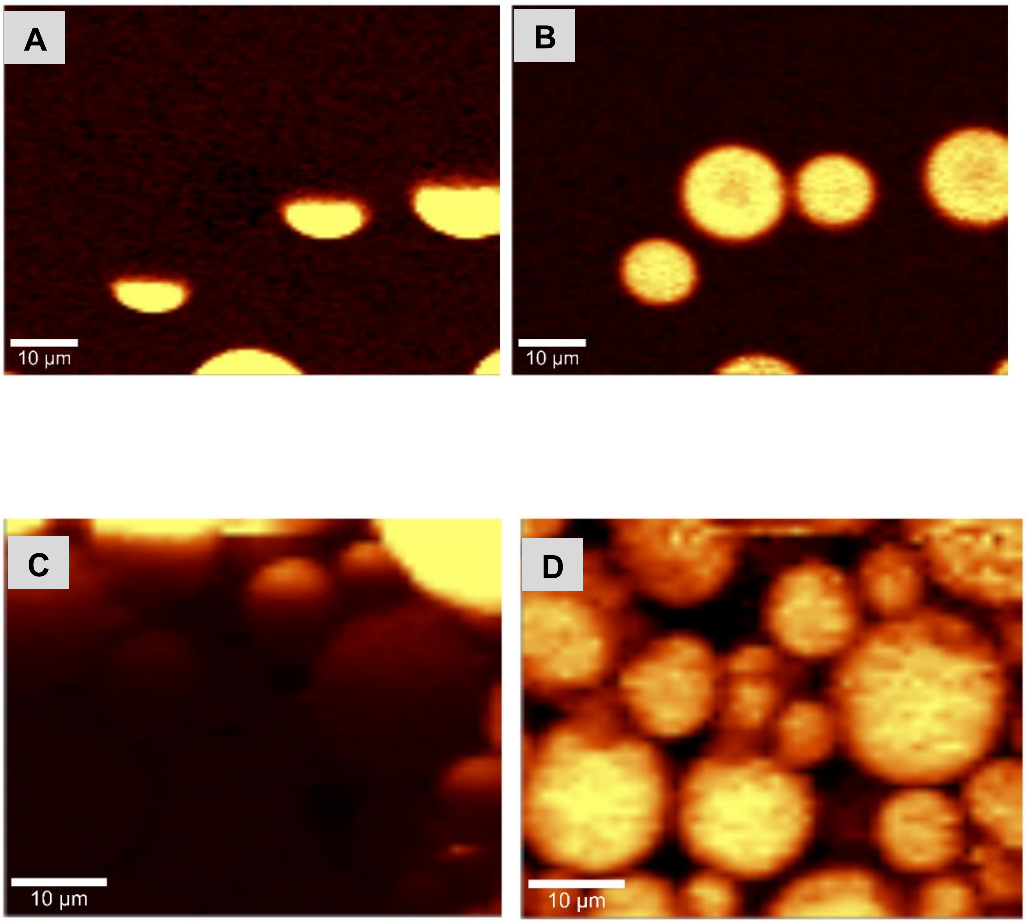

Figure 9: Confocal Raman microscopy images of DSEs scanned at high power (15 mW) showing evidence of possible photo-bleaching of β-carotene. A & C= β-carotene Raman channels, B & D= safflower oil Raman channels.
